# ZnO Catalyzed Efficient Synthesis of Some New 2-Substituted-4,6-diarylpyrimidines

**DOI:** 10.5402/2012/242569

**Published:** 2012-10-15

**Authors:** K. L. Ameta, Biresh Kumar, Nitu S. Rathore

**Affiliations:** Department of Chemistry, Faculty of Arts, Science and Commerce, Mody Institute of Technology & Science, Lakshmangarh 332311, Rajasthan, India

## Abstract

A simple and efficient protocol is developed for the synthesis of 2-substituted-4,6-diarylpyrimidines from one-pot three-component reaction of 4′-hydroxy-3′,5′-dinitro substituted chalcones, S-benzylthiouronium chloride (SBT), and heterocyclic secondary amines (morpholine/pyrrolidine/piperidine) in the presence of 15 mol% of ZnO as a heterogeneous catalyst. The present methodology offers several advantages such as being a simple procedure as well as providing excellent yields, and short reaction time. The catalyst is inexpensive, stable, and can be easily recycled and reused for several cycles with consistent activity.

## 1. Introduction 

Since many decades, bioactive heterocyclic compounds are one of the main topics of interest for the medicinal chemists as it displays a number of pharmacological activities. Nitrogen, sulphur, and oxygen containing five- and six-membered heterocyclic compounds have occupied enormous significance in the field of medicinal chemistry. The multicomponent reactions (MCRs) have emerged as an efficient and powerful tool in modern synthetic organic chemistry allowing the facile creation of several new bonds in a one-pot reaction. Therefore, in the last decade, research in academia and industry has increasingly emphasized the use of MCRs as well as domino reaction sequences for a broad range of products [1, 2]. Due to the atom economy, convergent character, and simplicity of one-pot procedures, multicomponent condensation reactions (MCRs) have an advantageous position among other reactions. The discovery and development of novel MCRs is receiving a growing interest from industrial chemistry research groups and represents a new challenge for organic chemists and to the basic understanding of organic chemistry itself [3].

Recently, intensive studies have been focused on the development of catalytic systems owing to their importance in synthetic organic chemistry. One of the most attractive synthetic strategies favoured by organic chemists is the use of heterogeneous catalyst in increasing the efficiency of a wide range of organic synthesis. Heterogeneous catalysis is being used in the fine chemical industries because of the need for more environmental friendly production technology. This tendency is assisted by the availability of catalytic materials and modern techniques of creating and investigating specific active sites on catalyst surfaces [4, 5]. Metal oxides exhibit both Lewis acid and Lewis base character at their surface [6].

The basic skeleton of chalcones possesses an *α*, *β*-unsaturated carbonyl group, used as the starting material for the synthesis of variously substituted different sized heterocycles of physiological importance like antiviral [7], antimalarial [8–10], antileishmanial [11, 12], antioxidant [13], anticancer [14, 15], and antimicrobial [16]. Pyrimidines and its derivatives are known as an important class of heterocyclic compounds in the pharmaceutical industry as well as in synthetic Chemistry [17]. On the other hand, the pyrimidines unit is a privileged heterocyclic motif that forms the core of a large family of nucleic acids and natural products with strong bioactivity profiles and significant structural properties [18]. 

**Scheme 1:**
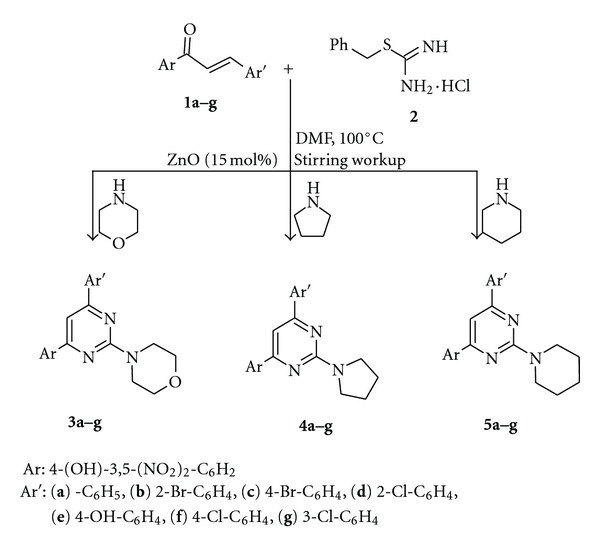
Synthesis of the title compounds.

Keeping in view of diverse biological activities of pyrimidines, it was thought to construct a novel system which may combine these bioactive rings together in a single molecular framework to see the additive effects towards their biological activities. Hence, as a part of our ongoing program to develop efficient and robust methods for the preparation of biologically relevant compounds [19], we have developed a facile and efficient catalytic approach for the multicomponent one-pot synthesis of novel 2-substituted-4,6-diarylpyrimidines (Scheme 1).

S-benzylthiouronium chloride (SBT) [20] and dicyandiamide (DDA) [21] have emerged from our laboratory team as versatile reagents for the continued synthesis of 2, 4, 6-tri substituted pyrimidines from *α*, *β*-unsaturated ketones and heterocyclic secondary amines. Consequently, facile synthesis of substituted pyrimidines was carried out using SBT, *α*, *β*-unsaturated ketones and organic bases under microwave irradiation with [22] or without [23] solvents. 

 To the best of our knowledge, there is no report available in the literature describing the use of ZnO as a catalyst for the synthesis of pyrimidine derivatives. ZnO is very reactive because it offers higher surface area and low coordinating sites. The surface area of the catalyst increases tremendously when size decreases and is responsible for the higher catalytic activity [24]. The effectiveness of the process was studied by comparing the results obtained with and without catalyst under normal conditions. Herein, we wish to report a novel synthesis of 4,6-diaryl-2-(4-morpholinyl/1-pyrrolidinyl/1-piperidinyl)-pyrimidines using ZnO as an efficient, nontoxic, reusable, and commercially available catalyst.

## 2. Results and Discussion

As a part of our ongoing research interest aimed at developing new synthetic strategies for the bioactive heterocyclic framework, the reaction of chalcones, S-benzylthiouronium chloride, and heterocyclic secondary amines was examined in the presence of catalytic amount (15 mol%) of ZnO under stirring condition at 100°C for 6 hour to give 2-substituted-4,6-diarylpyrimidine derivatives up to quantitative yields. A conceivable mechanism for the formation of the product would be as follows: the ZnO particle facilitates the Michael addition type coupling through Lewis acid sites (Zn^2+^) coordinated to the enone functionality. On the other hand, ZnO particles can activate SBT so that deprotonation of the N–H bond occurs in the presence of Lewis basic sites (O^2−^). As a result, the formation of 4,6-diaryl-2-benzylthiopyrimidines proceeds by activation of reactants through both Lewis acids and basic sites of ZnO catalyst and then nucleophilic substitution occurs by heterocyclic secondary amines and afforded 4,6-diaryl-2-(4-morpholinyl/1-pyrrolidinyl/1-piperidinyl)-pyrimidines. The catalyst could be recovered easily by solvent extraction of the product from the reaction mixture. For this, ethyl acetate was used; the aqueous layer containing the ZnO particles could be used for the next cycle.

The reaction was optimized for various reaction parameters such as temperature, solvent, and catalyst loading. The chalcones remain unconsumed when the reaction was done at room temperature. The effect of temperature on the yield of product was monitored from 60 to 120°C (Table 1, entry **3b**). However, no further increase in the yield was obtained by increasing the temperature from 100 to 120°C. Hence 100°C was chosen as optimum reaction temperature.

An attempt to catalyze the reaction in the absence of solvent resulted in very low yields (Table 2, entry **3b**). Among the various solvents studied, DMF was found to be the best solvent giving maximum yield of desired product.

Catalyst concentration was optimized by varying its concentration from 5 to 20 mol% (Table 3, entry **3b**). Increase in the product yield was observed form 5 to 20 mol% of catalyst amount. Hence 15 mol% was considered as an optimum catalyst concentration. The transformations of the reacting species were also confirmed by the spectroscopic studies. In FTIR, the disappearance of band at 1680–1710 cm^−1^ was due to the carbonyl group of chalcone, the appearance of band at 1595–1630 cm^−1^ due to cyclization, and in ^1^H NMR the appearance of multiplet at *δ* 3.40–3.90 for the –CH_2_–N–CH_2_– of morpholine/pyrrolidine/piperidine. 

**Figure 1:**
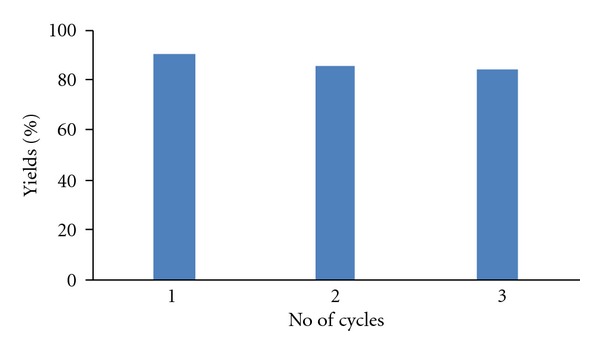
Recyclability of ZnO catalyst.

Reusability is one of the important properties of this catalyst. In this study, the catalyst was recovered by filtration from the reaction mixture and reused during three consecutive runs without any apparent loss of activity for the same reaction Figure 1. In order to confirm the effective involvement of ZnO catalyst during transformation, we also performed the experiment under conventional heating without using catalyst [25].

## 3. Conclusion

We have developed a simple, economic, eco-friendly and highly efficient synthetic strategy for exclusive synthesis of 2-substituted-4,6-diarylpyrimidines using inexpensive, recyclable, and commercially available catalyst. On the other hand, ZnO is remarkably easier to use, nonhazardous, inexpensive, and works under mild conditions.

## 4. Experimental Section

### 4.1. General

 The reaction mixtures were stirred magnetically. Chemicals were purchased from Sigma-Aldrich, Merck, and used without further purification. ^1^H- and ^13^C NMR spectra were recorded using (CDCl_3_) on 400 MHz ^1^H NMR spectrometer Bruker AV III. The chemical shifts are denoted in *δ* units (ppm) relative to TMS (*δ* = 0.00) for protons ^1^H: s (singlet), t (triplet), and m (multiplet). Melting points (°C) were measured in open glass capillaries using a Veego (VMP-MP) melting point apparatus and are uncorrected. Infrared spectra (*ν*, cm^−1^) were recorded on a Perkin-Elmer spectrophotometer model RX I. Elemental analyses (C, H, N) were in full agreement with the proposed structures within ±0.5% of the theoretical values on a Carlo Erba 1108 analyzer. Monitoring the reactions and checking the purity of the final products were carried out by thin layer chromatography (TLC) using silica gel precoated aluminum sheets (Merck, 60–120 mesh) and visualization with ultraviolet light (UV) at 365 nm and 254 nm. 

#### 4.1.1. Procedure for the Synthesis of 4,6-Diaryl-2-(4-morpholinyl/1-pyrrolidinyl/1-piperidinyl) Pyrimidines

The starting compounds (chalcones) were prepared by the Claisen-Schmidt condensation [16]. A mixture of substituted chalcone (0.002 mol), SBT (0.0022 mol), heterocyclic secondary amine (0.0024 mol), and ZnO (15 mol%) in DMF (10 mL) was taken in a 100 mL round bottomed flask and heated at 100°C temperature for 6 h under vigorous stirring. After completion of the reaction as indicated by TLC [Benzene: Ethylacetate, 9 : 1v/v], reaction mixture was cooled at room temperature and filtered to separate the catalyst. Removal of the excess of solvent under reduced pressure gave crude solid which on recrystallization afforded the title products.

#### 4.1.2. Spectral Analysis of 4,6-Diaryl-2-(4-morpholinyl)-pyrimidines **3a–g**

**(3a)**. Yield 86%, mp 105–107°C. IR (KBr): *ν* 3460, 3115, 1598, 1481, 1253 cm^−1^. ^1^H NMR (400 MHz, CDCl_3_): *δ* 3.45–3.65 (m, 4H, –CH_2_–N–CH_2_–), 3.70–3.90 (m, 4H, –CH_2_–O–CH_2_–), 6.80–7.30 (m, 5H, Ar-H), 7.70 (s, 1H), 8.48–8.62 (m, 2H), 12.05 (s, 1H, Ar-OH) ppm. ^13^C NMR (400 MHz, CDCl_3_): *δ* 47.52, 69.51, 123.80, 126.45, 128.65, 130.23, 143.46, 148.53, 150.50, 155.90 ppm. MS *m/z* 423 (M^+^). Anal. calcd for C_20_H_17_N_5_O_6_: C 56.74; H 4.05; N 16.54. Found: C 56.70, H 3.99, N 16.49.

**(3b)**. Yield 90%, mp 165–167°C. IR (KBr): *ν* 3469, 3125, 1600, 1478, 1252, 861 cm^−1^. ^1^H NMR (400 MHz, CDCl_3_): 3.50–3.70 (m, 4H, –CH_2_–N–CH_2_–), 3.75–3.90 (m, 4H, –CH_2_–O–CH_2_–), 6.80–7.15(m, 4H, Ar-H), 7.80 (s, 1H), 8.25–8.55 (m, 2H), 12.00 (s, 1H, Ar-OH) ppm. ^13^C NMR (400 MHz, CDCl_3_): *δ* 47.45, 68.60, 123.85, 125.80, 128.80 130.23, 142.45, 148.50, 151.12, 155.90, 159.53 ppm MS *m/z* 502 (M^+^). Anal. calcd For C_20_H_16_BrN_5_O_6_: C 47.83; H 3.21; N 13.94. Found: C 47.81, H 3.18, N 13.92.

**(3c).** Yield 89%, mp 100–102°C. IR (KBr): *ν* 3462, 3116, 1595, 1475, 1250, 863 cm^−1^. ^1^H NMR (400 MHz, CDCl_3_): *δ* 3.55–3.70 (m, 4H, –CH_2_–N–CH_2_–), 3.60–3.88 (m, 4H, –CH_2_–O–CH_2_–), 6.88–7.45 (m, 4H, Ar-H), 7.78 (s, 1H), 8.45–8.63 (m, 2H), 12.10 (s, 1H, Ar-OH) ppm. ^13^C NMR (400 MHz, CDCl_3_): *δ* 47.42, 68.90, 124.81, 126.15, 127.80 131.25, 141.15, 148.20, 152.10, 155.35, 158.50 ppm. MS *m/z* 502 (M^+^). Anal. calcd For C_20_H_16_BrN_5_O_6_: C 47.83; H 3.21; N 13.94. Found: C 47.85, H 3.17, N 13.90.

**(3d)**. Yield 88%, mp 160–162°C. IR (KBr): *ν* 3465, 3115, 1598, 1471, 1253, 810 cm^−1^. ^1^H NMR (400 MHz, CDCl_3_): *δ* 3.45–3.75 (m, 4H, –CH_2_–N–CH_2_–), 3.65–3.90 (m, 4H, –CH_2_–O–CH_2_–), 6.80–7.20 (m, 4H, Ar-H), 7.75 (s, 1H), 8.40–8.60 (m, 2H), 11.90 (s, 1H, Ar-OH) ppm. ^13^C NMR (400 MHz, CDCl_3_): *δ* 47.45, 68.60, 123.85, 125.80, 128.80 130.23, 142.45, 148.50, 151.12, 155.90, 159.53 ppm. MS *m/z* 457 (M^+^). Anal. calcd For C_20_H_16_ClN_5_O_6_: C 52.47; H 3.52; N 15.30. Found: C 52.43, H 3.51, N 15.28.

**(3e)**. Yield 86%, mp 97–99°C. IR (KBr): *ν* 3473, 3118, 1593, 1480, 1256, 821 cm^−1^. ^1^H NMR (400 MHz, CDCl_3_): *δ* 3.50–3.65 (m, 4H, –CH_2_–N–CH_2_–), 3.70–3.92 (m, 4H, –CH_2_–O–CH_2_–), 6.85–7.25 (m, 4H, Ar-H), 7.70 (s, 1H), 8.45–8.62 (m, 2H), 9.80 (s, 1H, Ar-OH), 12.00 (s, 1H, Ar-OH) ppm. ^13^C NMR (400 MHz, CDCl_3_): *δ* 47.05, 68.15, 124.80, 125.95, 129.15, 131.45, 144.23, 148.15, 150.36, 155.90, 159.95 ppm. MS *m/z* 439 (M^+^). Anal. calcd For C_20_H_17_N_5_O_7_: C 54.67; H 3.90; N 15.94. Found: C 54.55, H 3.81, N 15.78.

**(3f)**. Yield 89%, mp 136–138°C. IR (KBr): *ν* 3468, 3111, 1605, 1479, 1258, 818 cm^−1^. ^1^H NMR (400 MHz, CDCl_3_): *δ* 3.50–3.78 (m, 4H, –CH_2_–N–CH_2_–), 3.70–3.95 (m, 4H, –CH_2_–O–CH_2_–), 6.65–7.05 (m, 4H, Ar-H), 7.55 (s, 1H), 8.42–8.61 (m, 2H), 12.10 (s, 1H, Ar-OH) ppm. ^13^C NMR (400 MHz, CDCl_3_): *δ* 47.05, 68.95, 123.48, 126.95, 128.35, 131.55, 144.25, 148.86, 150.85, 156.25, 159.25 ppm. MS *m/z* 457 (M^+^). Anal. calcd For C_20_H_16_ClN_5_O_6_: C 52.47; H 3.52; N 15.30. Found: C 52.41, H 3.49, N 15.29.

**(3g)**. Yield 90%, mp 125–127°C. IR (KBr): *ν* 3460, 3114, 1600, 1472, 1258, 820 cm^−1^. ^1^H NMR (400 MHz, CDCl_3_): *δ* 3.55–3.78 (m, 4H, –CH_2_–N–CH_2_–), 3.72–3.95 (m, 4H, –CH_2_–O–CH_2_–), 6.65–7.08 (m, 4H, Ar-H), 7.58 (s, 1H), 8.42–8.61 (m, 2H), 12.08 (s, 1H, Ar-OH) ppm. ^13^C NMR (400 MHz, CDCl_3_): *δ* 47.05, 68.95, 124.48, 125.95, 127.35, 131.55, 145.25, 147.52, 150.85, 156.65, 159.25 ppm. MS *m/z* 457 (M^+^). Anal. calcd For C_20_H_16_ClN_5_O_6_: C 52.47; H 3.52; N 15.30. Found: C 52.40, H 3.45, N 15.23.

#### 4.1.3. Spectral Analysis of 4,6-Diaryl-2-(1-pyrrolidinyl)-pyrimidines) **4a–g**

**(4a)**. Yield 88%, mp 81–83°C. IR (KBr): *ν* 3462, 3115, 1598, 1475, 1266 cm^−1^. ^1^H NMR (400 MHz, CDCl_3_): *δ* 1.70–1.90 (m, 4H, –CH_2_–CH_2_–), 3.72–3.90 (m, 4H, –CH_2_–N–CH_2_–), 6.80–7.20 (m, 4H, Ar-H), 7.35 (s, 1H), 8.41–8.63 (m, 2H), 11.80 (s, 1H, Ar-OH) ppm. ^13^C NMR (400 MHz, CDCl_3_): *δ* 25.23, 27.68, 47.80, 123.15, 126.26, 128.30, 130.13, 143.50, 148.45, 155.30 ppm. MS *m/z* 407 (M^+^). Anal. calcd for C_20_H_17_N_5_O_5_: C 58.97; H 4.21; N 17.19. Found: C 58.93, H 4.18, N 17.15.

**(4b)**. Yield 89%, mp 150–152°C. IR (KBr): *ν* 3460, 3112, 1595, 1477, 1258, 868 cm^−1^. ^1^H NMR (400 MHz, CDCl_3_): *δ* 1.70–1.93 (m, 4H, –CH_2_–CH_2_–), 3.61–3.79 (m, 4H, –CH_2_–N–CH_2_–), 6.90–7.25 (m, 4H, Ar-H), 7.61 (s,1H), 8.41–8.62 (m, 2H), 12.01 (s, 1H, Ar-OH) ppm. ^13^C NMR (400 MHz, CDCl_3_): *δ* 25.25, 27.96, 46.45, 124.45, 126.85, 129.80, 130.50, 142.50, 147.22, 148.87, 155.58, MS *m/z* 486 (M^+^). Anal. calcd For C_20_H_16_BrN_5_O_5_: C 49.40; H 3.32; N 14.40. Found: C 49.38, H 3.28, N 14.35.

**(4c)**. Yield 86%, mp 161-162°C. IR (KBr): *ν* 3468, 3121, 1603, 1478, 1265, 865 cm^−1^. ^1^H NMR (400 MHz, CDCl_3_): *δ* 1.71–1.98 (m, 4H, –CH_2_–CH_2_–), 3.52–3.75 (m, 4H, –CH_2_–N–CH_2_–), 6.90–7.35 (m, 4H, Ar-H), 7.70 (s, 1H), 8.40–8.60 (m, 2H), 11.95 (s, 1H, Ar-OH) ppm. ^13^C NMR (400 MHz, CDCl_3_): *δ* 25.56, 27.25, 47.00, 124.56, 126.12, 129.11, 130.50, 141.00, 146.00, 148.56, 155.01. ppm MS *m/z* 486 (M^+^). Anal. calcd For C_20_H_16_BrN_5_O_5_: C 49.40; H 3.32; N 14.40. Found: C 49.37, H 3.31, N 14.37.

**(4d)**. Yield 86%, mp 175-176°C. IR (KBr): *ν* 3470, 3120, 1596, 1481, 1261, 815 cm^−1^. ^1^H NMR (400 MHz, CDCl_3_): *δ* 1.70–1.90 (m, 4H, –CH_2_–CH_2_–), 3.52–3.70 (m, 4H, –CH_2_–N–CH_2_–), 7.00–7.35 (m, 4H, Ar-H), 7.70 (s, 1H), 8.35–8.55 (m, 2H), 12.00 (s, 1H, Ar-OH) ppm. ^13^C NMR (400 MHz, CDCl_3_): *δ* 25.05, 27.89, 47.78, 125.05, 126.56, 129.55, 130.02, 140.15, 146.85, 147.95, 155.85, 159.36 ppm. MS *m/z* 441 (M^+^). Anal. calcd For C_20_H_16_ClN_5_O_5_: C 54.37; H 3.65; N 15.85. Found: C 54.35, H 3.60, N 15.81.

**(4e)**. Yield 90%, mp 78–80°C. IR (KBr): *ν* 3455, 3110, 1600, 1475, 1258, 818 cm^−1^. ^1^H NMR (400 MHz, CDCl_3_): *δ* 1.70–1.92 (m, 4H, –CH_2_–CH_2_–), 3.55–3.73 (m, 4H, –CH_2_–N–CH_2_–), 6.85–7.25 (m, 4H, Ar-H), 7.75 (s, 1H), 8.41–8.60 (m, 2H), 9.56 (s, 1H, Ar-OH), 12.01 (s, 1H, Ar-OH) ppm. ^13^C NMR (400 MHz, CDCl_3_): *δ* 25.05, 27.80, 47.06, 124.00, 126.45, 128.05, 130.54, 139.25, 146.02, 147.30, 156.26, 159.80 ppm. MS *m/z* 423 (M^+^). Anal. calcd For C_20_H_17_N_5_O_6_: C 56.74; H 4.05; N 16.54. Found: C 56.65, H 3.92, N 16.42.

**(4f)**. Yield 88%, mp 125–127°C. IR (KBr): *ν* 3470, 3135, 1615, 1469, 1245, 819 cm^−1^. ^1^H NMR (400 MHz, CDCl_3_): *δ* 1.65–1.88 (m, 4H, –CH_2_–CH_2_–), 3.58–3.78 (m, 4H, –CH_2_–N–CH_2_–), 6.85–7.20 (m, 4H, Ar-H), 7.65 (s, 1H), 8.35–8.55 (m, 2H), 12.00 (s, 1H, Ar-OH) ppm. ^13^C NMR (400 MHz, CDCl_3_): *δ* 25.05, 27.80, 47.12, 124.45, 126.85, 127.00, 129.50, 141.00, 146.01, 147.86, 156.14, 159.15 ppm. MS *m/z* 441 (M^+^). Anal. calcd For C_20_H_16_ClN_5_O_5_: C 54.37; H 3.65; N 15.85. Found: C 54.34, H 3.63, N 15.81. 

**(4g)**. Yield 90%, mp 102–104°C. IR (KBr): *ν* 3475, 3131, 1612, 1470, 1248, 822 cm^−1^. ^1^H NMR (400 MHz, CDCl_3_): *δ* 1.68–1.86 (m, 4H, -CH_2_-CH_2_-), 3.58–3.80 (m, 4H, –CH_2_–N–CH_2_–), 6.82–7.20 (m, 4H, Ar-H), 7.65 (s, 1H), 8.33–8.52 (m, 2H), 12.02 (s, 1H, Ar-OH) ppm. ^13^C NMR (400 MHz, CDCl_3_): *δ* 25.08, 27.82, 47.12, 123.45, 125.80, 127.05, 129.55, 141.06, 147.05, 147.86, 156.20, 159.10 ppm. MS *m/z* 441 (M^+^). Anal. calcd For C_20_H_16_ClN_5_O_5_: C 54.37; H 3.65; N 15.85. Found: C 54.30, H 3.60, N 15.80.

#### 4.1.4. Spectral Analysis of 4,6-Diaryl-2-(1-piperidinyl)-pyrimidines) **5a–g**

**(5a)**. Yield 89%, mp 116–118°C. IR (KBr): *ν* 3460, 3119, 1599, 1479, 1259 cm^−1^. ^1^H NMR (400 MHz, CDCl_3_): *δ* 1.40–1.62 (m, 6H, –(CH_2_)_2_–), 3.40–3.71 (m, 4H, –CH_2_–N–CH_2_–), 3.79 (s, 3H, Ar-OCH_3_), 6.90–7.20 (m, 4H, Ar-H), 7.65 (s, 1H), 8.35–8.60 (m, 2H), 11.25 (s, 1H, Ar-OH) ppm. ^13^C NMR (400 MHz CDCl_3_): *δ* 25.20, 27.35, 47.55, 123.75, 128.54, 130.55, 143.85, 148.75, 150.45, 155.90 ppm. MS *m/z* 421 (M^+^). Anal. calcd for C_21_H_19_N_5_O_5_: C 59.84; H 4.54; N 16.62. Found: C 59.80, H 4.49, N 16.68.

**(5b).** Yield 86%, mp 171–173°C. IR (KBr): *ν* 3465, 3111, 1605, 1481, 1261, 855 cm^−1^. ^1^H NMR (400 MHz, CDCl_3_): *δ* 1.30–1.58 (m, 6H, –(CH_2_)_3_–), 3.45–3.72 (m, 4H, –CH_2_–N–CH_2_–), 6.60–7.00 (m, 4H, Ar-H), 7.65 (s, 1H), 8.30–8.55 (m, 2H), 11.90 (s, 1H, Ar-OH) ppm. ^13^C NMR (400 MHz, CDCl_3_): *δ* 25.42, 27.40, 47.75, 124.00, 129.15, 130.05, 143.15, 148.00, 148.85, 150.04, 155.00 ppm. MS *m/z* 400 (M^+^). Anal. calcd For C_21_H_18_BrN_5_O_5_: C 50.41; H 3.63; N 14.00. Found: C 50.38, H 3.61, N 13.98.

**(5c)**. Yield 89%, mp 152–1154°C. IR (KBr): *ν* 3460, 3115, 1610, 1490, 1255, 861 cm^−1^. ^1^H NMR (400 MHz, CDCl_3_): *δ* 1.30–1.50 (m, 6H, –(CH_2_)_2_–), 4.40–4.71 (m, 4H, –CH_2_–N–CH_2_–), 6.90–7.35 (m, 4H, Ar-H), 7.70 (s, 1H), 8.45–8.62 (m, 2H), 11.65 (s, 1H, Ar-OH) ppm. ^13^C NMR (400 MHz, CDCl_3_): *δ* 25.45, 27.40, 47.80, 124.65, 129.00, 129.95, 143.58, 147.05, 148.95, 151.15, 155.65 ppm. MS *m/z* 400 (M^+^). Anal. calcd For C_21_H_18_BrN_5_O_5_: C 50.41; H 3.63; N 14.00. Found: C 50.37, H 3.65, N 14.02.

**(5d)**. Yield 87%, mp 201–203°C. IR (KBr): *ν* 3469, 3118, 1603, 1479, 1253, 802 cm^−1^. ^1^H NMR (400 MHz, CDCl_3_): 1.40–1.65 (m, 6H, –(CH_2_)_2_–), 3.42–3.65 (m, 4H, –CH_2_–N–CH_2_–), 6.95–7.35 (m, 4H, Ar-H), 7.66 (s, 1H), 8.42–8.65 (m, 2H), 12.00 (s, 1H, Ar-OH) ppm. ^13^C NMR (400 MHz, CDCl_3_): *δ* 25.55, 27.05, 47.80, 124.15, 129.45, 130.02, 143.15, 147.00, 148.23, 151.01, 156.35 ppm. MS *m/z* 455 (M^+^). Anal Anal. calcd For C_21_H_18_ClN_5_O_5_: C 55.33; H 3.98; N 15.36. Found: C 55.31, H 3.95, N 15.33.

**(5e)**. Yield 87%, mp 100–102°C. IR (KBr): *ν* 3468, 3121, 1596, 1471, 1261, 820 cm^−1^. ^1^H NMR (400 MHz, CDCl_3_): *δ* 1.48–1.68 (m, 6H, –(CH_2_)_3_–), 3.48–3.71 (m, 4H, –CH_2_–N-CH_2_–), 6.92–7.25 (m, 4H, Ar-H), 7.65 (s, 1H), 8.42–8.60 (m, 2H), 8.56 (s, 1H, Ar-OH), 12.10 (s, 1H, Ar-OH) ppm. ^13^C NMR (400 MHz, CDCl_3_): *δ* 25.50, 27.23, 47.56, 124.15, 130.01, 131.55, 143.50, 147.48, 148.01, 150.45, 156.85, 159.50 ppm. MS *m/z* 437 (M^+^). Anal. calcd For C_21_H_19_N_5_O_6_: C 57.66; H 4.38; N 16.01. Found: C 57.51, H 4.25, N 15.92.

**(5f)**. Yield 86%, mp 201–203°C. IR (KBr): *ν* 3464, 3118, 1601, 1468, 1258, 809 cm^−1^. ^1^H NMR (400 MHz, CDCl_3_): *δ* 1.35–1.53 (m, 6H, –(CH_2_)_3_–), 3.48–3.70 (m, 4H, –CH_2_–N–CH_2_–), 6.80–7.30 (m, 4H, Ar-H), 7.60 (s, 1H), 8.35–8.58 (m, 2H), 12.00 (s, 1H, Ar-OH) ppm. ^13^C NMR (400 MHz, CDCl_3_): *δ* 25.21, 27.23, 47.15, 124.56, 130.00, 131.35, 141.52, 146.14, 148.45, 151.56, 156.42, 159.23 ppm. MS *m/z* 455 (M^+^). Anal. calcd For C_21_H_18_ClN_5_O_5_: C 55.33; H 3.98; N 15.36. Found: C 55.31, H 3.95, N 15.30. 

**(5g)**. Yield 88%, mp 160–162°C. IR (KBr): *ν* 3466, 3120, 16012, 1465, 1258, 810 cm^−1^. ^1^H NMR (400 MHz, CDCl_3_): *δ* 1.35–1.55 (m, 6H, –(CH_2_)_3_–), 3.45–3.71 (m, 4H, –CH_2_–N–CH_2_–), 6.78–7.32 (m, 4H, Ar-H), 7.65 (s, 1H), 8.35–8.58 (m, 2H), 12.02 (s, 1H, Ar-OH) ppm. ^13^C NMR (400 MHz, CDCl_3_): *δ* 25.21, 27.25, 47.20, 123.50, 131.02, 132.30, 140.51, 145.18, 147.41, 150.55, 157.40, 160.20 ppm. MS *m/z* 455 (M^+^). Anal. calcd For C_21_H_18_ClN_5_O_5_: C 55.33; H 3.98; N 15.36. Found: C 55.28, H 3.92, N 15.25.

## Figures and Tables

**Table 1:** Effect of temperature for the synthesis of 2-substituted-4,6-diarylpyrimidine^a^.

Entry	Temperature (°C)	Yield (%)
1	Room temp	Nil
2	60	30
3	80	60
4	100	90
5	120	90

^a^Reaction conditions: chalcone (0.002 mol), SBT (0.0022 mol), heterocyclic secondary amine (0.0024 mol), and ZnO catalyst (15 mol%) in DMF (10 mL) at 100°C temperature for 6 h.

**Table 2:** Effect of solvent for the synthesis of 2-substituted-4,6-diarylpyrimidine^a^.

Entry	Solvent	Yield (%)
1	None	20
2	H_2_O	30
3	NMP	68
4	DMSO	75
5	DMF	90

^a^Reaction conditions: chalcone (0.002 mol), SBT (0.0022 mol), heterocyclic secondary amine (0.0024 mol), and ZnO catalyst (15 mol%) in DMF (10 mL) at 100°C temperature for 6 h.

**Table 3:** Effect of catalyst loading for the synthesis of 2-substituted-4,6-diarylpyrimidine^a^.

Entry	Catalyst (mol%)	Yield (%)
1	5	45
2	10	60
3	15	90
4	20	90

^a^Reaction conditions: chalcone (0.002 mol), SBT (0.0022 mol), heterocyclic secondary amine (0.0024 mol), and ZnO catalyst in DMF (10 mL) at 100°C temperature for 6 h.
